# Transitioning to adulthood: Are conventional benchmarks as protective today as they were in the past?

**DOI:** 10.1016/j.ssresearch.2024.102981

**Published:** 2024-03

**Authors:** Christal Hamilton, Zachary Parolin, Jane Waldfogel, Christopher Wimer

**Affiliations:** a School of Public Policy, University of Connecticut, USA; b Department of Social and Political Sciences, Bocconi University, Milan, Italy; c School of Social Work, Columbia University, USA; d Center on Poverty and Social Policy, Columbia University, USA

**Keywords:** Poverty, Social policy, Young adults

## Abstract

More young adults in the United States are studying beyond high school and working full-time than in the past, yet young adults continue to have high poverty rates as they transition to adulthood. This study uses longitudinal data on two cohorts of young adults from the 1979 and 1997 National Longitudinal Study of Youth to assess whether conventional benchmarks associated with economic success—gaining an education, finding stable employment, and delaying childbirth until after marriage—are as predictive of reduced poverty today as they were in the past. We also explore differences in the protective effect of the benchmarks by race/ethnicity, gender, and poverty status while young. We find that, on average, the benchmarks associated with economic success are as predictive of reduced poverty among young adults today as they were for the prior generation; however, demographics and features of the economy have contributed to higher poverty rates among today's young adults.

## Highlights

• Benchmarks of success are as predictive of reduced poverty today as in the past.
• Achieving more benchmarks increases the likelihood of reduced young adult poverty.
• Being Black and poor as a youth explain young adult poverty more than some benchmarks.
• Economic changes over time contribute to higher poverty rates for young adults today.

## 1 Introduction

Young adulthood is an important time in the life course when access to opportunities and achievements are crucial to later life success. Research highlights that achieving certain benchmarks in young adulthood, namely graduating from high school, maintaining a full-time job, and delaying childbirth until marriage, is associated with a higher likelihood of economic success and a reduced likelihood of being in poverty (Wang and Wilcox 2017). Indeed, one study found that 98 percent of adults who achieve these benchmarks avoid poverty (Haskins 2013).

Over the past 30 years, young adults in the United States (US) have progressively met these benchmarks. Young adults are not only graduating from high school, but are increasingly likely to attain higher education. Rates of college completion have substantially risen across generations for all income groups (Bailey and Dynarski 2011). Young adult employment rates—particularly among female young adults—have also increased in recent years (Sironi and Furstenberg 2012). Further, while more young adults are having children before marriage by ages 28–34 relative to the prior generation, 67 percent of millennials have delayed childbirth until marriage or are unmarried and childless (Wang and Wilcox 2017).

Yet despite increasingly meeting these benchmarks of success, young adults today continue to experience poverty and economic insecurity as they progress to adulthood. Not only do young adults today have the highest poverty rates among all age groups, but unlike other age groups, the poverty rate among young adults has moderately risen since the 1960s (Wimer et al., 2020). Young adults in recent years also face challenges that hinder their economic security including higher debt, fewer labor market opportunities, a decline in real annual earnings, and high living costs (Bialik and Fry 2019; Sum and McLaughlin 2011). Considering societal changes and the new challenges faced by young adults today, one can reasonably question whether the traditional benchmarks for economic success are as effective today as they were in the past in predicting young adult success.

This study assesses whether gaining an education, finding stable employment, and delaying childbirth until after marriage are as predictive of reduced poverty today as they were in the past. Specifically, we use data from the 1979 and 1997 cohorts of the National Longitudinal Study of Youth (NLSY) to test whether young adults today (1997 cohort) who meet more of these benchmarks have different likelihoods of being poor at age 30 compared to young adults in the past (1979 cohort). We also explore differences by race/ethnicity, gender, and poverty status while young.

We find achieving the benchmarks is as predictive of reduced poverty among today's generation of 30-year-olds as it was for the previous generation. Though the association between partnering before having children and poverty has decreased slightly over time, the association between having at least a high school education or being consistently employed and poverty have either increased or remained the same. We also find no significant differences across generations in the association between meeting the three benchmarks and poverty. Nevertheless, our results show that other factors contribute to higher poverty rates among today's young adults relative to the earlier cohort. In particular, we find that demographic characteristics and factors associated with the economy have contributed to higher poverty rates among today's young adults.

## 2 Background

### 2.1 The transition to adulthood and changing economic circumstances of young adults

The transition to adulthood—the process of moving from adolescence to adulthood— is an important life transition. Success in young adulthood encompasses multiple dimensions including good physical health, psychological and emotional well-being, ethical behavior, life skills, and healthy family and social relationships (Scales et al., 2016), as well as the dimensions we focus on here: completing school, leaving home, securing employment and starting one's own family (Settersten Jr and Ray, 2010). Whereas fifty years ago young adults had achieved economic stability and formed families by their early twenties, this process has become longer and more gradual (Arnett 2007, Williamson and Côté, 2022). Young adults today are delaying school completion, leaving their parental home, marriage, and childbirth, thereby taking longer to attain economic and psychological autonomy compared to their counterparts in the 1960s and 1970s (Arnett 2006, Settersten Jr and Ray, 2010; Williamson and Côté, 2022). Postsecondary enrollment rates in the US have increased from 26 percent in 1970 to 41 percent in 2019 (De Brey et al., 2021). In 1960 about 29 percent of young adults aged 18–29 lived with their parents, but this proportion increased to 46 percent by 2019 (Fry et al., 2020). Similarly, in 1968 around 45 percent of young adults ages 18–24 were married (Furstenberg 2010) and around one percent were cohabitating compared to seven and nine percent, respectively, in 2018 (United States Census Bureau 2018).

Several structural and cultural factors have contributed to the changes in the transition to adulthood in the US. More women are pursuing tertiary education and entering the workforce, perceptions on marriage and cohabitation have changed, and advances in contraception and women's rights have given women increased fertility choices (Danziger and Rouse, 2007; Edin and Kefalas 2011). Another contributing factor is the transformation in economic conditions in the US over the past fifty years. In the years following World War II, the US economy was prosperous with numerous well-paying and secure jobs available, particularly in the manufacturing sector. Employment and wages grew rapidly during this time and young adults were able to secure high-paying jobs that provided benefits but required minimal education (Danziger and Gottschalk 1995, Danziger and Rouse, 2007; Settersten Jr and Ray, 2010; Williamson and Côté, 2022). The economic security these jobs provided allowed many young adults to sequentially achieve the milestones associated with adulthood, that is (completing high school, securing a job, living independently, getting married and establish families) soon after high school (Williamson and Côté, 2022). By the mid-1970s, however, the period of economic growth had ended and globalization, deindustrialization, and technical changes led to a worsening labor market (Danziger and Gottschalk 1995; Danziger and Ratner 2010; Sum and McLaughlin 2011, Williamson and Côté, 2022). Manufacturing and unionized jobs decreased as the economy became more dependent on skilled labor (Danziger and Ratner 2010; Sum et al., 2011). At the same time, income inequality increased because of differences in earnings between highly educated and low educated workers, as well as a decline in inflation adjusted minimum wages (Danziger and Ratner 2010; Sum et al., 2011; Sum and McLaughlin 2011).

While all age groups were impacted by the economic changes in the US in the 1980s, young adults were especially hit hard because these changes decreased their employment and financial stability (Danziger and Rouse, 2007; Sum and McLaughlin 2011, Williamson and Côté, 2022), which in turn affected their ability to sequentially meet various milestones during the transition through adulthood (Williamson and Côté, 2022). Young adults today face increased difficulty in finding and maintaining full-time employment (Farber 2008; Sum et al., 2011, Williamson and Côté, 2022), and many experience underemployment. The annual earnings of young adults have also decreased in recent years—particularly among young men (Danziger and Rouse, 2007; Sum et al., 2011; Sum and McLaughlin 2011, Williamson and Côté, 2022). Compared to the earnings of young adult men in 1978, real annual earnings for young adult men in 2009 declined by about 15 percent (Sum and McLaughlin 2011), with declines being largest among those with less than a high school degree (Danziger and Rouse, 2007; Sum and McLaughlin 2011). In contrast, annual earnings among young adult women increased by 17 percent during the same period, driven largely by increases in real annual earnings for those women with a college degree or more (Sum and McLaughlin 2011). Moreover, the percent of young adult men with earnings below the poverty line has increased in recent years (Danziger and Rouse 2007). These labor market changes have had real consequences for young adults—particularly young adult men—because they have resulted in young adults taking longer to earn enough income to support a family and achieve self-sufficiency (Danziger and Rouse, 2007; Williamson and Côté, 2022).

The greater demand for skilled labor and employment uncertainties since 1980 have also impacted young adults’ education and family formation decisions. As discussed above, the economic returns to education have significantly increased since the 1970s (Barrow and Rouse, 2011, Furstenberg 2010; Sironi and Furstenberg 2012; Sum and McLaughlin 2011). Young adults in the previous generation were able to have a decent income and quality of life with a high school degree. Today, post-secondary qualifications are needed to attain a high paying quality job and economic security. As a result, many young adults—including an increased number of women—are staying in school longer to improve their job prospects and economic status, thereby delaying family formation (Arnett 2000; Danziger and Rouse, 2007; Mason and Jensen 1995).

The economic changes since the mid-1970s have not only altered the transition to adulthood for young adults but have also led to differences in transitions by income and racial groups. Researchers highlight that one's path through the transition to adulthood is largely determined by family background (Furstenberg 2008, Guldi et al., 2007) and that young adults from disadvantaged backgrounds are less likely to successfully transition to adulthood (Furstenberg 2008; Osgood et al., 2019). Higher-income and highly-educated parents are able to use their income and wealth, as well as their social and cultural capital to ensure their children are able to successfully transition to adulthood and achieve the various benchmarks necessary for economic stability (Osgood et al., 2019). As a result, young adults from advantaged backgrounds are more likely to have the skills and resources needed to attain a college degree (Furstenberg 2008; Guldi et al., 2007), to secure well-paying jobs, and are more likely to be civically engaged (Scales et al., 2016). These young adults are also more likely to attain markers associated with adulthood such as home ownership (Charles and Hurst 2002; Furstenberg 2008; Schoeni and Ross 2008). Conversely, young adults from disadvantaged backgrounds are less likely to have the same economic and cultural capital from their parents to enter college and persist to degree attainment, or to find well-paying full-time jobs (Fuligni and Hardway, 2004; Furstenberg 2008; Terriquez and Gurantz 2015). Further, since the 1980s employment prospects for African Americans have declined, resulting in increased unemployment and lower wages for young adults among this group (Corcoran and Matsudaira, 2005). This in turn, may have contributed to changes in family formation as declines in employment activities have been linked to decreased marriage rates and increased out of wedlock births (Dorn and Hanson 2019).

Recent research highlights that educational attainment, full-time employment, and marriage before childbirth increases the likelihood of being in the middle class and escaping poverty in adulthood, including for those from racial minority backgrounds (Reeves et al., 2015, Sawhill 2018; Wang and Wilcox 2017; Wilcox and Wang 2022). Among households achieving these three benchmarks, only two percent were poor, while 55 percent were middle income or higher. Conversely, 76 percent of those who had failed to achieve any of these benchmarks were in poverty and less than five percent were in the middle class or higher (Haskins and Sawhill, 2009). Young adults today are more educated than their predecessors and are increasingly delaying marriage and childbirth, yet they have the highest poverty rates of any age group in the US population. Young adults also face challenges that make it difficult to secure jobs paying adequate wages to ensure their economic stability or support a family (Settersten Jr and Ray, 2010; Smeeding and Phillips, 2002). Though postsecondary enrollment rates have increased, only 39 percent of young adults in 2020 had attained a college degree or higher (Irwin et al., 2021). Additionally, while educational attainment has increased, young adults today are earning less than their predecessors. Young adult men in 2002 with less than a college degree earned less than comparably educated young adult men in 1975, with young men with some college earning about $3500 a year less than in 1975 (Settersten Jr and Ray, 2010). In 2004, more young adults were earning wages below the federal poverty line than in the 1970s, and since then have had consistently higher poverty rates than the older adult population (Wimer et al., 2020). Rising costs of living, an inadequate minimum wage, wage stagnation, and increased job instability mean that many young adults—particularly those with a high school degree or less—face greater difficulty to achieve economic stability (Danziger and Ratner 2010, Settersten Jr and Ray, 2010).

Given the high poverty rates among young adults despite meeting benchmarks related to economic success, in this study we assess whether the benchmarks of success are less predictive of reduced poverty today than they were in the past. Specifically, we examine whether young adults today who have at least a high school degree, are consistently employed, and marry before childbearing have different likelihoods of being in poverty at age 30 compared to young adults in the past. We also explore differences by gender, race/ethnicity, and poverty status while young.

## 3 Data and methods

### 3.1 Data

We used data from the 1979 and 1997 cohorts of the National Longitudinal Study of Youth (NLSY). The NLSY is a nationally representative longitudinal survey administered by the U.S. Bureau of Labor Statistics. The first NLSY cohort originally comprised 12,686 respondents who were ages 14–22 when interviewed in 1979. After dropping two sub-samples by 1990, the current 1979 cohort includes 9964 respondents. The second NLSY cohort was initially surveyed in 1997 and included 8984 respondents ages 12–17 (US Bureau of Labor Statistics, 2021). Since their initial interviews, survey participants in each cohort have been re-interviewed either annually or bi-annually as they progress through adulthood. Designed to gather information on labor market activities and major life events, the NLSY surveys contain high-quality measures of education, employment, income, benefit receipt, and other metrics necessary to characterize economic profiles throughout adulthood and over time. The NLSY also allowed us to observe the same young adults from their teenage years through adulthood at age 30.

### 3.2 Sample

We limited our sample to adults at age 30. For adults not observed precisely at age 30, we take their first recorded observation after age 30 but before 35. In practice, 58 percent of adults are included at age 30, 28 percent are included at age 31, and the remaining 14 percent at ages 32–34. Next, we excluded adults with missing information on the main dependent variable, poverty status. Our final analytical sample size comprised 15,063 young adults—8321 from the 1979 cohort and 6742 from the 1997 cohort.

### 3.3 Measures

#### 3.3.1 Benchmarks

We included three measures for young adults’ attainment of benchmarks of success that are typically associated with reduced likelihood of poverty in the prior literature. Having a high school degree or more was a dichotomous measure equal to one if a young adult had attained a high school degree or more education prior to age 30. Consistent employment was an indicator for whether young adults worked at least 35 hours per week for at least three (observed) years between the ages of 25 and 29; we also set this indicator to one if a young adult was enrolled in school full-time, or was enrolled in school and working part-time at least three (observed) years between the ages of 25 and 29. Married or cohabitating before children was an indicator equal to one if young adults were married or cohabitating before having children prior to age 30, or were childless prior to age 30, and equal to zero if they had children outside of marriage or cohabitation before age 30. We also created indicators for whether young adults had all three measures, two measures, or just one measure of success.

As part of sensitivity analyses, we also created alternative specifications for each of our three benchmarks. Alternative educational attainment specifications that we examined included four binary indicators for whether a young adult had a high school degree (or GED), a high school degree and some college, a Bachelor's degree, and a post-Bachelor's degree. Our alternative measure for consistent employment was an indicator for whether a young adult worked at least 35 h per week for at least three consecutive years between the ages of 25 and 29 (thus setting those who were in full-time education or combining education and employment to zero). For partnering before birth, we created a dichotomous variable equal to one only if a young adult was married before having children prior to age 30 or if they had no children, and equal to zero if they had children without marrying (even if they were cohabiting).

#### 3.3.2 Economic well-being

Our primary measure of economic well-being is poverty status around age 30. The poverty indicator provided in the NLSY is similar to the Official Poverty Measure (OPM), but with a few important differences. First, the standard OPM excludes benefits from the SNAP program in its calculation of family resources. In the NLSY, however, benefits from the Supplemental Nutrition Assistance Program (SNAP, often referred to as food stamps) are typically included as part of family resources. We are unable to consistently remove only SNAP benefits from a family's income to create a pre-SNAP measure of income; we thus leave the benefits in the resources calculation while acknowledging that it leads to a modified version of the OPM. We do not view this as a limitation, since our preferred definition of resources is one that includes SNAP benefits.

Second, the standard OPM does not account for tax credits or tax liabilities. Given the rise of the Earned Income Tax Credit (EITC) and the Child Tax Credit (CTC) throughout recent decades, however, accounting for tax credits is particularly important in assessments of poverty. We simulate tax liabilities and tax credits using TAXSIM. As state identifiers are not available in the NSLY, we only simulate federal income taxes and forego simulation of state income taxes. We view the exclusion of state income taxes as a minor limitation, as federal income taxes are the dominant share of tax liabilities and credits for nearly all tax-filing households. We add (or subtract) the simulated tax credits and liabilities to the respondent's family income and recalculate the respondent's poverty status accordingly, still using the OPM poverty thresholds.

The NLSY does not measure several income components that are not easily computable. These include the value of housing subsidies, WIC and the School Lunch Program, energy assistance from LIHEAP, and other smaller-scale social programs. Our income definition is thus not a complete measure of resources, but does likely capture the bulk of post-tax, post-transfer resources for the majority of households in the sample.

Third, the resource-sharing unit for the standard OPM is related family members within the household. In the NLSY, however, the unit of analysis varies slightly between the 1979 and 1997 cohorts. In the 1979 cohort, the incomes of non-married partners are not included as part of the respondent's family resources before 1994. In the 1997 cohort, non-married partners are treated as spouses with respect to income sharing and are included as part of the respondent's family resources. Our preference is to include non-married, cohabitating partners, particularly given the rise of this family structure in recent decades. We therefore reconstruct a family income measure that includes income from non-married partners from 1990 to 1993 in the 1979 NLSY cohort; this information is provided directly by the NLSY from 1994 onwards.

In sum, the modifications to resource measurement and the resource sharing unit provide us with a post-tax, post-transfer measure of family income (including non-married partners) that is assessed relative to the standard OPM thresholds. We refer to this measure as a measure of poverty, but we explicitly acknowledge that this poverty measure differs from the standard OPM.

#### 3.3.3 Controls

We included controls in our models for demographic and socioeconomic characteristics such as sex, race/ethnicity, family income, and living arrangements, which would be expected to affect young adults' risk of poverty. Sex was a dichotomous measure equal to one if a young adult was female and zero if male. Racial/ethnic controls included indicators for whether a young adult was non-Hispanic Black, Hispanic, or non-Hispanic White or another race/ethnicity. We also included indicators for region of residence, whether a young adult was poor when young (ages 14–17), and whether they lived with parents at age 30. Further, we included a continuous measure of respondents’ number of own children.

#### 3.3.4 Missing data

In addition to NLSY item non-response, in some cases respondents have missing information on the three benchmark measures because they were not surveyed during the years we use to measure attainment of these benchmarks. Additionally, poverty status has a somewhat high level of missingness in the NLSY because of missingness in family income measures. Statistical tests indicate several demographic factors were associated with the probability of having missing data on these benchmarks, indicating that the data are not missing at random. For example, race/ethnicity. We therefore also included dichotomous indicators in our analytical models for whether young adults had missing data on their educational attainment, employment status, and their poverty status when young. Though controlling for missingness in our models may not fully compensate for potential biases, this approach is generally preferred to dropping cases with missing data (Hargrove, 2018).

### 3.4 Analytical approach

We first present descriptive statistics on the prevalence of benchmarks of success for 30-year-olds for the two NLSY cohorts. We also present trends in the differences in poverty rates among individuals meeting the benchmarks of success**.** The second part of our analytical approach includes a series of linear probability models to estimate the conditional associations between the benchmarks of success and poverty at age 30. Our baseline model uses the following equation:

(1) $$y_{i}=\beta_{1}{\left(Education\right)}_{i}+\beta_{2}{\left(Employment\right)}_{i}+\beta_{3}{\left(DelayedChild\right)}_{i}+\varepsilon_{i}$$

where *y*_*i*_ represents the poverty status of each young adult. The three explanatory variables are dichotomous indicators for each benchmark, that is, whether a young adult achieved a high school degree or more, was consistently employed, or had delayed childbirth until after marriage or cohabitation. Next, we run two additional models controlling first for demographic characteristics (sex, race/ethnicity, and whether in poverty when young), then adding controls for living with parents, the number of own children, and region of residence. All estimation models include age effects to control for differences in the likelihood of poverty that may occur due to being slightly older than age 30.

In addition to estimating models separately for each NLSY cohort, we estimated models that pooled the data from both cohorts and interacted each benchmark with a cohort indicator. We also estimated models to examine the likelihood of being in poverty if a young adult achieved, one, two, or all three benchmarks and compare these across generations, as well as models by race/ethnicity, gender, and poverty status while young to determine heterogeneous effects.

Finally, we apply a Kitagawa-Blinder-Oaxaca decomposition analysis (Blinder 1973; Kitagawa 1955; Oaxaca 1973) to estimate whether differences in poverty between the two NLSY cohorts relate to differences in the prevalence of benchmarks, and/or the returns to those benchmarks. The decomposition analysis also helps us to estimate what poverty rates might look like among the 30-year-olds in the 1997 sample if they matched the characteristics and had the same returns to those characteristics, as in the 1979 sample. Specifically, we apply a two-fold decomposition that breaks down how the change in poverty rates between the two cohorts can be attributed to changes in composition (such as shifts in the share of adults meeting our benchmarks of interest) or changes in how those compositional features are associated with poverty (such as how having a high school degree may be more or less associated with poverty in the 1997 cohort versus the 1979 cohort).

Note that throughout we use the terms “effects” or “returns” to refer to the estimated association between meeting conventional benchmarks of success and poverty. We use these terms not to imply a causal link between any given benchmark and later economic well-being, but simply to characterize the associations between the benchmarks and poverty observed in our data and how those change over time.

## 4 Findings

### 4.1 Descriptive findings

**Fig. 1:**
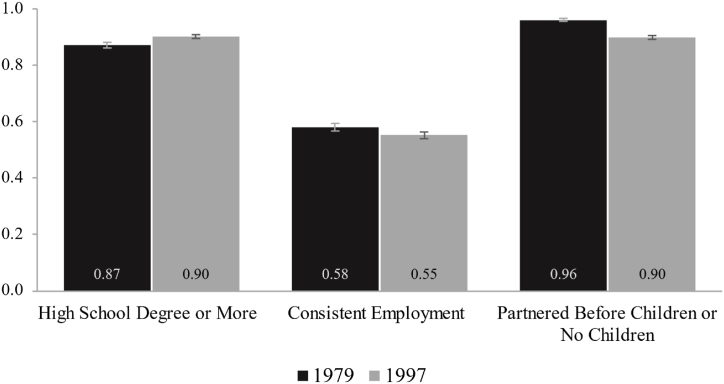
Descriptive Characteristics Around Age 30 by NLSY Cohort. *Notes*: Authors' analyses using data from the 1979 and 1997 NLSY cohorts.

Fig. 1 displays descriptive statistics for each benchmark among 30-year-old adults by NLSY cohort. The 30-year-olds in the 1997 cohort are slightly more likely to have a high school degree or more and slightly less likely to be consistently employed. The primary difference between the two cohorts relates to partnering before childbearing: the more recent cohort is slightly less likely to be childless or to have partnered before childbirth (90%) compared to the 1979 cohort (96%). But overall, the most striking finding from Fig. 1 is the high rate of benchmark attainment in both cohorts. We provide more detailed descriptive characteristics for the study sample around age 30 by NLSY cohort in Appendix Table A1.

**Table 1:** Estimated effect of benchmarks on poverty around age 30 b y NLSY cohort.

	Model 1		Model 2		Model 3	
	1979	1997	1979	1997	1979	1997
High School Degree or More	−0.20***	−0.26***	−0.18***	−0.22***	−0.18***	−0.21***
	(0.02)	(0.02)	(0.02)	(0.02)	(0.02)	(0.02)
Consistent Employment	−0.14***	−0.12***	−0.12***	−0.12***	−0.12***	−0.12***
	(0.01)	(0.01)	(0.01)	(0.01)	(0.01)	(0.01)
Partnered Before Child or No Child	−0.23***	−0.18***	−0.15***	−0.13***	−0.15***	−0.13***
	(0.04)	(0.02)	(0.04)	(0.02)	(0.04)	(0.02)
Poverty When Young			0.11***	0.14***	0.12***	0.14***
			(0.02)	(0.02)	(0.02)	(0.02)
Female			0.01	−0.02**	0.03**	−0.02**
			(0.01)	(0.01)	(0.01)	(0.01)
Black			0.14***	0.14***	0.14***	0.14***
			(0.02)	(0.01)	(0.02)	(0.01)
Hispanic			0.06**	0.02	0.05*	0.01
			(0.02)	(0.01)	(0.02)	(0.01)
Living with Parent(s) at age 30					0.08**	0.04***
					(0.02)	(0.01)
Number of Children at Age 30					−0.02	0.00
					(0.01)	(0.00)
Region of residence					X	X
Constant	0.82***	0.39*	0.52**	0.36*	0.42*	0.45*
	(0.15)	(0.18)	(0.16)	(0.17)	(0.17)	(0.18)
Observations	4822	6742	4822	6742	4822	6742

*Notes*: Authors' analyses using data from the 1979 and 1997 NLSY cohorts. Standard errors are presented in parentheses; *p < 0.05, **p < 0.01, ***p < 0.001. OPM poverty rate: 10.9 percent in 1979 cohort, 13.8 percent in 1997 cohort. All models control for age, missing poverty status when young, missing employment status, and missing education attainment.

**Fig. 2:**
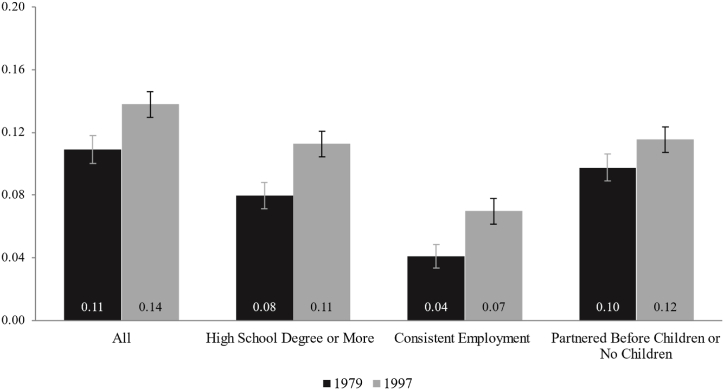
Poverty Rate by Benchmark Around Age 30. *Notes*: Authors' analyses using data from the 1979 and 1997 NLSY cohorts.

We display poverty rates overall and by benchmark status for each cohort in Fig. 2. The first panel shows that overall poverty rates increased modestly between the two cohorts: the average poverty rate among 30-year-olds in the 1979 cohort was 11%, compared to 14% among the 1997 cohort. Among individuals with a high school degree or more and those with full time work, poverty rates for the 1997 cohort were three percentage points higher than for the 1979 cohort, while poverty rates among those who were childless or partnered before having children differed only slightly between the two cohort (10% for the 1979 cohort and 12% in the 1997 cohort). Although the increases in poverty are small, all the differences in poverty rates between the cohorts shown in Fig. 2 were statistically significant. In Appendix Table A2, we provide poverty rates by detailed descriptive characteristics.

**Table 2:** Estimated effects of benchmarks on poverty around age 30: Including cohort indicator and interactions between benchmarks and cohort.

	Model 1	Model 2	Model 3
High School Degree or More	−0.20***	−0.18***	−0.18***
	(0.02)	(0.02)	(0.02)
High school degree or more X 1997 Cohort	−0.07*	−0.05	−0.05
	(0.03)	(0.03)	(0.03)
Consistent Employment	−0.14***	−0.13***	−0.12***
	(0.01)	(0.01)	(0.01)
Consistent Employment X 1997 Cohort	0.01	0.01	0.010
	(0.01)	(0.01)	(0.01)
Partnered before child or no child	−0.23***	−0.15***	−0.15***
	(0.04)	(0.04)	(0.04)
Partnered before child or no child X 1997 Cohort	0.06	0.03	0.03
	(0.04)	(0.04)	(0.04)
1997 Cohort	0.02	0.03	0.02
	(0.04)	(0.04)	(0.04)
Poverty when young		0.12***	0.13***
		(0.01)	(0.01)
Female		−0.00	0.00
		(0.01)	(0.01)
Black		0.14***	0.14***
		(0.01)	(0.01)
Hispanic		0.04***	0.03**
		(0.01)	(0.01)
Living with Parent(s) at age 30			0.05***
			(0.01)
Number of children at age 30			−0.00
			(0.00)
Region of residence			0.00
Constant	0.70***	0.49***	0.47***
	(0.12)	(0.12)	(0.13)
Observations	11,564	11,564	11,564

Notes: Authors' analyses of 1979 and 1997 NLSY cohort data. Standard errors are presented in parentheses; *p < 0.05, **p < 0.01, ***p < 0.001. OPM poverty rate: 10.9 percent in 1979 cohort, 13.8 percent in 1997 cohort. All models control for age, missing poverty status when young, missing employment status, and missing education attainment.

### 4.2 Empirical estimates

In Table 1, we present the results from our estimates assessing how the given benchmarks—high school degree or more, full-time work, and partnered before children or childless—are associated with a 30-year-old's poverty status in the 1979 and 1997 cohorts. The results show that obtaining a high-school degree or more has a larger effect on poverty than any other benchmark or sociodemographic characteristic. In our base model—before controlling for other demographic or socioeconomic characteristics—we find that the association between obtaining a high-school degree or more and poverty was larger for the 1997 cohort than for the 1979 cohort. When including controls for age sex, race/ethnicity, and an indicator for whether the 30-year-old lived in poverty between the ages of 14 and 17, the reduction in poverty associated with having a high-school degree or more decreases for both cohorts but remains higher for the 1997 cohort (18 vs 22 percentage points, respectively). In our full model specification, which includes additional controls for whether young adults live with their parents, their number of own children, and region of residence at age 30, the association between a high-school degree and reduced poverty remain the same for the 1979 cohort but decreases by one percentage point for the 1997 cohort.

Similar to obtaining at least a high school degree, being consistently employed is associated with a reduced likelihood of poverty for both cohorts. For our base model, being consistently employed is associated with only a slightly larger reduction in poverty for the 1979 cohort (14 percentage points) compared to the 1997 cohort (12 percentage points) and decreases slightly for the 1979 cohort when we control for sociodemographic characteristics and region of residence. In our full model specification, the association between this benchmark and reduced poverty is the same for both cohorts (12 percentage points).

Our results show the most changes across cohorts and models with respect to partnering before childbirth or not having children. In our base model, this benchmark is associated with a 23 percentage-point reduction in poverty for the 1979 cohort and a 18 percentage-point reduction for the 1997 cohort. When controlling for sociodemographic characteristics and region of residence, the reduction in poverty associated with being childless or partnering before childbirth becomes smaller and remains slightly higher for the 1979 cohort (15 percentage points) than for the 1997 cohort (13 percentage points).^1^

The results from our full model specification also reveal that being poor while young, being Black, and living with parents at age 30 are associated with an increased likelihood of being poor for both cohorts. A 30-year-old in the 1997 cohort who experienced poverty during their youth is 14 percentage points more likely to be poor, independent of education, sex, race/ethnicity, marriage status, or the other socio-demographic controls included. This effect is larger than the estimated association for the 1979 cohort (12 percentage points). Being female for the 1979 cohort is associated with a three-percentage point increase in poverty and a two-percentage point decrease in poverty for the 1997 cohort. Conversely, the associations between being Black or living with parents at age 30 and poverty remained the same over time (14 percentage points). The association between living with parents at age 30 and poverty is four percentage points lower for the 1997 cohort than the 1979 cohort (4 vs. 8 percentage points).

In addition to running our analyses by NLSY cohort, we also estimated models where we pooled the data from the two cohorts and included a binary indicator equal to one if a young adult was in the 1997 cohort and equal to zero if they were in the 1979 cohort. This indicator was included as a main effect and also interacted with each benchmark. The results from these models—presented in Table 2—reveal that there were no statistically significant differences between the associations between the benchmarks and poverty across the two cohorts.

Next, we examined the likelihood of being in poverty if a young adult achieved, one, two or all three benchmarks and compared these across generations. We present the results from these models in Table 3. As expected, we find that achieving more benchmarks of success is associated with a larger decrease in the likelihood of being poor. We also find that the associations were lower for the 1997 cohort than for the 1979 cohort. Relative to not achieving any of the benchmarks of success, achieving just one benchmark reduces a young adult's likelihood of being in poverty by 25 percentage points for the 1979 cohort and by 23 percentage points for the 1997 cohort. Achieving two benchmarks of success reduces the likelihood of being in poverty by 50 percentage points for young adults in the 1979 cohort, and by 42 percentage points for the 1997 cohort. Finally, for young adults in the 1997 cohort, attaining all three benchmarks reduces the likelihood of being in poverty by 53 percentage points, a five-percentage-point lower reduction compared to young adults from the previous cohort.

**Table 3:** Estimated effects of achieving varied number of benchmarks on poverty around age 30.

	Model 1		Model 2		Model 3	
	1979	1997	1979	1979	1997	1979
One benchmark	−0.31***	−0.28***	−0.25**	−0.23***	−0.25**	−0.23***
	(0.08)	(0.05)	(0.08)	(0.05)	(0.08)	(0.05)
Two benchmarks	−0.59***	−0.51***	−0.50***	−0.42***	−0.50***	−0.42***
	(0.07)	(0.05)	(0.07)	(0.05)	(0.07)	(0.05)
Three benchmarks	−0.69***	−0.62***	−0.59***	−0.53***	−0.58***	−0.53***
	(0.07)	(0.05)	(0.07)	(0.05)	(0.07)	(0.05)
Poverty when young			0.12***	0.14***	0.12***	0.14***
			(0.02)	(0.02)	(0.02)	(0.02)
Female			0.01	−0.03***	0.03*	−0.03***
			(0.01)	(0.01)	(0.01)	(0.01)
Black			0.13***	0.14***	0.12***	0.13***
			(0.02)	(0.01)	(0.02)	(0.01)
Hispanic			0.05**	0.02*	0.05*	0.02
			(0.02)	(0.01)	(0.02)	(0.01)
Living with Parent(s) at age 30					0.08**	0.04***
					(0.02)	(0.01)
Number of children at age 30					−0.02	0.00
					(0.01)	(0.00)
Region of residence					X	X
Constant	0.96***	0.47**	0.66***	0.44*	0.57**	0.54**
	(0.16)	(0.18)	(0.18)	(0.18)	(0.18)	(0.18)
Observations	4822	6742	4822	6742	4822	6742

*Notes*: Authors' analyses using data from the 1979 and 1997 NLSY cohorts. Standard errors are presented in parentheses; *p < 0.05, **p < 0.01, ***p < 0.001. OPM poverty rate: 10.9 percent in 1979 cohort, 13.8 percent in 1997 cohort. All models control for age, missing poverty status when young, missing employment status, and missing education attainment.

### 4.3 Heterogeneous effects

Table 4 presents heterogenous effects of the benchmarks by gender, race, and youth poverty status for the 1979 and 1997 cohorts. These results indicate that the conclusion that the effects of the benchmarks on reducing poverty have not changed between cohorts is true not only for the population overall, but also when looking specifically at results for women, Black individuals, and those who experienced poverty when young. The results also show that the magnitude of the effects of the benchmarks differs by gender, race, and youth poverty status.

**Table 4:** Heterogeneous effects of benchmarks on poverty around age 30.

	High School Degree or More		Consistent Employment		Partnered Before Child or No Child	
	1979	1997	1979	1997	1979	1997
Men	−0.17**	−0.18***	−0.13***	−0.14***	0.02	−0.14***
	(0.02)	(0.03)	(0.02)	(0.01)	(0.06)	(0.04)
Women	−0.20***	−0.26***	−0.12***	−0.09***	−0.18***	−0.12***
	(0.03)	(0.04)	(0.02)	(0.02)	(0.04)	(0.02)
Black	−0.20***	−0.25***	−0.19***	−0.22***	−0.13*	−0.17***
	(0.05)	(0.04)	(0.04)	(0.02)	(0.05)	(0.03)
Hispanic	−0.33***	−0.19***	−0.19***	−0.12**	−0.21	−0.06
	(0.06)	(0.04)	(0.04)	(0.02)	(0.10)	(0.03)
White or other race/ethnicity	−0.15***	−0.20***	−0.11***	−0.10***	−0.08	−0.12***
	(0.02)	(0.03)	(0.01)	(0.01)	(0.05)	(0.03)
Not poor when young	−0.18***	−0.22***	−0.11***	−0.10***	−0.17***	−0.10***
	(0.02)	(0.03)	(0.01)	(0.01)	(0.04)	(0.02)
Poor when young	−0.17**	−0.15***	−0.27***	−0.23***	−0.04	−0.23***
	(0.05)	(0.04)	(0.05)	(0.03)	(0.09)	(0.04)

*Notes*: Authors' analyses using data from the 1979 and 1997 NLSY cohorts. The top panel presents results for gender groups, the middle panel for racial groups and the 1979 cohort, and the bottom panel presents results by poverty status when young. OPM poverty rate: 10.9 percent in 1979 cohort, 13.8 percent in 1997 cohort. All models control for age, demographics, region of residence, missing poverty status when young, missing employment status, and missing education attainment.

The first panel presents heterogenous effects for females and males at age 30. For the 1979 cohort, men who had at least a high school degree or were consistently employed were 17 and 13 percentage points, respectively, less likely to be in poverty. Women who obtained a high school degree or more, however, were 20 percentage points less likely to be poor and those who were consistently employed were 12 percentage points less likely to be poor. While partnering before children was not associated with poverty status for men, for women this benchmark reduced the poverty among women by 18 percentage points. For the 1997 cohort, the association between having a high school degree or more was larger for women than for men (26 vs. 18 percentage points), while the effect of consistent employment and partnering before children on poverty reduction was lower for women than men.

The second panel shows the effects for adults by racial groups. In both cohorts, the effects of all three benchmarks on poverty is greater for Black adults than adults who are White or of another race/ethnicity, with effects of the benchmarks being generally larger in the 1997 cohort. For the 1979 cohort, compared to adults who are White or other race/ethnicity, the effects of having a high school degree or more or being consistently employed on poverty were both larger for Hispanic adults. For the in the 1997 cohort, the effect of having a high school degree or more on poverty was lower for Hispanics than for adults who are White or of another race/ethnicity.

The third panel in Table 4 presents the effects of the benchmarks on poverty by youth poverty status. For both cohorts, having a high school degree or more has a smaller effect on poverty for adults who were poor when young compared to adults who did not grow up in poverty. Conversely, for both cohorts the association between poverty and being consistently employed and partnering before children was larger for the adults who were poor when young.

### 4.4 Decomposition analyses

Table 5 presents results from our Kitagawa-Blinder-Oaxaca decomposition. The top panel of Table 5 displays the poverty rates among young adults in the two cohorts, showing that the poverty rate increases from 10.90% to 13.79% across the two cohorts, a difference of 2.89 percentage points. The prevalence column indicates how changes in the prevalence of the given indicator contribute to that 2.89 percentage point increase in poverty, while the returns column indicates how differences in the returns to a given indicator (i.e., differences in the association of that indicator with poverty) contribute to the increase in poverty.

**Table 5:** Kitagawa-oaxaca-blinder decomposition of differences in poverty rates among 30-Year-Olds In 1979; 1997 samples.

	1979 Poverty Rate	1997 Poverty Rate	Difference
	10.90%	13.79%	2.89 p.p.
	**Due to differences in prevalence**	**Due to differences in returns**	**Total**
**Benchmarks (all)**	**0.006**	**−0.033***(n.s.)*	**−0.027**
Education	−0.009	−0.050 *(n.s.)*	−0.059
Consistent Work	0.007	0.006 *(n.s.)*	0.013
Partnering before childbirth	0.009	0.011 *(n.s.)*	0.020
**Demographics (all)**	**0.008**	**−0.056**	**−0.048**
Female	0.000 *(n.s.)*	−0.052	−0.052
Black	0.004	0.001 *(n.s.)*	0.003
Hispanic	0.004	−0.003 *(n.s.)*	0.001
**Constant**		**0.105**	**0.105**
**Total**	0.014	0.015	0.029

*Notes*: Authors' analyses using data from the 1979 and 1997 NLSY cohorts. N.S. indicates that the coefficient is not statistically significant at the 95 percent confidence level.

The rows related to the “Benchmarks” demonstrate that the small changes in the prevalence of the benchmarks, led by changes in partnering before childbirth, contributed to a slightly higher poverty rate (0.6 percentage points) among the 1997 cohort relative to the 1979 cohort than would have been observed otherwise. Combined with higher shares of Black and Hispanic individuals in the 1997 cohort, observed demographic changes contributed to a 0.8 percentage point higher poverty rate in the 1997 cohort. Combined, these changes contributed to a 1.4 percentage point higher poverty rate in the 1997 cohort relative to the 1979 cohort. That 1.4 percentage points, however, explains less than half of the observed 2.89 percentage points, suggesting that most of the change in poverty rates was not due to changes in composition.

The results in the returns column validate this claim. Achieving a high school degree is associated with a lower likelihood of poverty in the 1997 cohort compared to the 1979 cohort, while partnership before childbirth is associated with slightly higher poverty for the more recent cohort. Overall, meeting the benchmarks is associated with a 3-percentage point lower likelihood of poverty for the 1997 cohort, though this difference is not statistically significant. The likelihood of poverty for women is also notably lower (5-percentage points) in the more recent cohort. Nonetheless, there exists a 10-percentage point increase in poverty for all in the 1997 cohort, independent of returns to the specific compositional features we have measured, captured in the model's constant. Put differently, all other factors not included in our models, including unobserved features of the broader economy, contribute to a large, positive increase in the likelihood of poverty more generally. As a result, young adults in the 1997 cohort still face higher rates of poverty than the 1979 cohort *despite* meeting the education, employment, and family benchmarks in greater numbers and *despite* those benchmarks being associated with lower rates of poverty in 1997. In all, around 1.5 percentage points of the overall 2.89 percentage point increase in poverty across cohorts is due to factors other than changes in the benchmarks or basic demographic features.

## 5 Discussion

Despite achieving benchmarks traditionally associated with economic success, young adults in the US today continue to have high rates of poverty compared to other age groups. This study assessed whether traditional benchmarks of success are less predictive of reduced poverty among young adults today than in the past. Utilizing data from the 1979 and 1997 cohorts of the NLSY, we employed multivariate linear probability models and the Kitagawa-Blinder-Oaxaca decomposition model to determine whether attaining at least a high school degree, having full-time employment, and being childless or partnering before childbirth were less likely to be associated with reduced poverty among young adults at age 30 today than they were in the past. In addition to our main specification, we also ran analyses by gender, race, and poverty status while young to identify heterogeneous effects.

Overall, our findings suggest that relative to 30-year-olds in the previous generation, meeting the benchmarks are not less predictive of reduced poverty among today's generation of 30-year-olds. Indeed, we found no statistically significant differences in the associations between meeting specific benchmarks for current 30-year-olds as compared to 30-year-olds in the previous generation. Additionally, achieving a higher number of benchmarks significantly increases the likelihood of reduced poverty for 30-year-olds today as it did in the past.

We also highlight the association between demographic characteristics and economic wellbeing. Previous studies show that socioeconomic background has a small positive impact on young adults’ ability to achieve economic self-sufficiency (200 percent above the poverty line) and that this association decreased across generations (Sironi and Furstenberg 2012). We find, however, that demographic characteristics—namely being Black and living in poverty as a youth—are more or as important in explaining poverty status at age 30 than having full time employment and partnering before having children. Similar to the results for the overall population, the benchmarks have not changed in their effectiveness at reducing poverty among women, Black young adults, and young adults who experienced childhood poverty. However, Black young adults and young adults who experienced childhood poverty remain more likely to be poor compared to non-Black young adults and young adults who grew up in non-poor households.

What then explains the higher risk of poverty among today's young adults? Our results indicate that other changes over time (such as changes in broader features of the economy), contribute to higher poverty rates for today's young adults than those in the past. Current economic conditions and higher costs of living could be making economic stability increasingly hard for young adults, who are more vulnerable than their older counterparts. Our results also provide support for Brady, Finnigan, and Hübgen's (2017) ‘prevalence and penalties’ framework for analyzing and understanding the risks of poverty. Similar to Brady et al. (2017), we find that the prevalence of not achieving the three benchmarks among young adults is low. Across 29 rich democracies Brady et al. (2017) also found, however, that the US had the highest penalties (greater probabilities of poverty associated with a given risk factor of poverty) associated with not completing high school and was among the top five countries with the highest penalties for single motherhood and unemployed households. The high poverty rates among young adults could therefore be due in some part to the very high penalties associated with not achieving the benchmarks in the US, which could be tied to the continued barriers faced by minority groups. Future research should explore the factors contributing to the high penalties related to the benchmarks examined in this study.

While this study provides important insights into the association between poverty and some of the benchmarks of success during young adulthood, it is not without limitations. The NLSY has somewhat high levels of missing data for some variables, including income and poverty. About 15 percent of our initial sample had missing information on our outcome of interest. As a result, we may not be identifying the true prevalence of poverty among young adults at age 30.

The persistence of high poverty rates among young adults, and more so among Black young adults and young adults who grew up in poverty—despite achieving the benchmarks of success—is a pressing concern, particularly given that economic stability during young adulthood can have long term implications for young adults’ future wellbeing, and the wellbeing of their children and society. Young adults, especially childless young adults, have generally been ignored as a policy target population. Yet policymakers can and should implement policies that improve young adult economic well-being. The findings from this and previous studies (Brady et al., 2017) highlight the need for policies that address the stagnating returns to higher education and improve the labor market outcomes of young adults. Establishing a livable minimum wage and increasing wages across educational levels can improve the economic stability of young adults. Additionally, addressing the rising cost of college tuition and providing more grant aid for postsecondary pursuits not only can help reduce the debt burden of young adults, but also can increase the likelihood of young adults from low-income and minority backgrounds achieving postsecondary success. Brady et al. (2017) showed that social welfare generosity moderates the penalties for low education and unemployment. Expanding young adult eligibility for social programs as well as providing greater income support to families with children can decrease economic hardship among young adults. The recent expansions of the childless portion of the Earned Income Tax Credit and the Child Tax Credit are steps in this direction because they provide supplemental income to young adults that helps decrease income volatility. Nevertheless, further research is urgently needed to identify factors in the labor market that are contributing to young adult poverty so that other policy solutions can be identified.

## CRediT authorship contribution statement

**Christal Hamilton:** Formal analysis, Project administration, Writing – original draft, Writing – review & editing, Conceptualization. **Zachary Parolin:** Conceptualization, Formal analysis, Methodology, Writing – original draft, Writing – review & editing. **Jane Waldfogel:** Conceptualization, Writing – original draft, Writing – review & editing, Supervision. **Christopher Wimer:** Supervision, Conceptualization, Funding acquisition, Project administration, Writing – original draft, Writing – review & editing.

## Figures and Tables

**Table A.1:** Detailed Descriptive Characteristics Around Age 30 by NLSY Cohort

	1979	1997
Age	30.7	30.6
Female	50.0	48.4
*Race/Ethnicity*		
White or other race/ethnicity	81.8	72.4
Black	12.4	14.8
Hispanic	5.8	12.7
Living with parent(s) at age 30	6.2	25.5
Number of children at age 30	25.1	96.0
Poverty when young	8.1	11.6
Poverty when young missing	58.2	41.2
*Region of Residence*		
Northeast	17.4	15.83
North Central	28.56	24.28
South	34.71	37.26
West	18	21.75
Region missing	1.33	0.88
*Benchmarks*		
Less than high school	12.0	7.8
High school degree only	42.5	32.4
Some college	21.8	25.0
Bachelor's degree	15.3	26.2
Post-Bachelor's degree	8.4	8.6
Education missing	1.0	2.3
Consistent employment	58.08	55.30
Consistent employment missing	0.95	7.22
Married before children	45.49	29.52
Cohabitating before children	4.72	19.46
*N*	4822	6742

*Note*: Authors' analyses using data from the 1979 and 1997 NLSY cohorts.

**Table A.2:** Poverty Rates by Detailed Descriptive Characteristics

	1979	1997
All	0.11	0.14
Poverty when young	0.29	0.34
Poverty when young missing	0.10	0.12
No high school degree	0.33	0.42
High school degree only	0.11	0.20
Some College	0.08	0.10
Bachelor's degree	0.01	0.04
Post-Bachelor's degree	0.03	0.03
Education missing	0.08	0.21
Consistent employment	0.04	0.07
Consistent employment missing	0.07	0.27
Married before kids	0.10	0.06
Cohabitating before kids	0.25	0.18

*Note*: Authors' analyses using data from the 1979 and 1997 NLSY cohorts.

**Table B.1:** Estimated Effects of Benchmarks on Poverty Around Age 30; Alternative Specifications of Educational Attainment *Added Individually into Main Model*

	Model 1		Model 2		Model 3	
	1979	1997	1979	1997	1979	1997
High School Degree or More	−0.20***	−0.26***	−0.18***	−0.22***	−0.18***	−0.21***
	(0.02)	(0.02)	(0.02)	(0.02)	(0.02)	(0.02)
Some College or More	−0.08***	−0.15***	−0.07***	−0.12***	−0.07***	−0.12***^a^
	(0.01)	(0.01)	(0.01)	(0.01)	(0.01)	(0.01)
Bachelor's Degree or More	−0.09***	−0.11***	−0.07***	−0.08***	−0.07***	−0.08***
	(0.01)	(0.01)	(0.01)	(0.01)	(0.01)	(0.01)
Post-Bachelor's Degree	−0.07***	−0.08***	−0.05***	−0.06***	−0.05***	−0.06***
	(0.01)	(0.01)	(0.01)	(0.01)	(0.01)	(0.01)
Other Benchmarks	X	X	X	X	X	X
Demographics			X	X	X	X
All Controls					X	X
Observations	4822	6742	4822	6742	4822	6742

*Notes*: Authors' analyses using data from the 1979 and 1997 NLSY cohorts. Standard errors are presented in parentheses. The symbols *, **, and *** denote *p* < 0.05, p < 0.01, and *p* < 0.001 respectively. Superscripts a, b, and c indicate that the p value for the difference between cohorts is statistically significant at the 0.1%, 1%, and 5% levels, respectively. OPM poverty rate: 10.9 percent in 1979 cohort, 13.8 percent in 1997 cohort. All models control for age, missing poverty status when young, missing employment status, and missing education attainment.

**Table B.2:** Estimated Effects of Benchmarks on Poverty Around Age 30; Alternative Specification of Consistent Employment

	Model 1		Model 2		Model 3	
	1979	1997	1979	1979	1997	1979
Consistent work	−0.14***	−0.12***	−0.12***	−0.12***	−0.12***	−0.12***
	(0.01)	(0.01)	(0.01)	(0.01)	(0.01)	(0.01)
High school degree or more	−0.20***	−0.26***	−0.18***	−0.22***	−0.18***	−0.21***
	(0.02)	(0.02)	(0.02)	(0.02)	(0.02)	(0.02)
Partnered before kids	−0.23***	−0.18***	−0.15***	−0.13***	−0.15***	−0.13***
	(0.04)	(0.02)	(0.04)	(0.02)	(0.04)	(0.02)
Poverty when young			0.11***	0.14***	0.12***	0.14***
			(0.02)	(0.02)	(0.02)	(0.02)
Female			0.01	−0.02**	0.03**	−0.02**
			(0.01)	(0.01)	(0.01)	(0.01)
Black			0.14***	0.14***	0.14***	0.14***
			(0.02)	(0.01)	(0.02)	(0.01)
Hispanic			0.06**	0.02	0.05*	0.01
			(0.02)	(0.01)	(0.02)	(0.01)
Living with parent(s) at age 30					0.08***	0.04***
					(0.02)	(0.01)
Number of children at age 30					−0.02	0.00
					(0.01)	(0.00)
Region of residence					X	X
Constant	0.82***	0.39*	0.52**	0.36*	0.42*	0.45*
	(0.15)	(0.18)	(0.16)	(0.17)	(0.17)	(0.18)
Observations	4822	6742	4822	6742	4822	6742

*Notes*: Authors' analyses using data from the 1979 and 1997 NLSY cohorts. Standard errors are presented in parentheses. The symbols *, **, and *** denote *p* < 0.05, p < 0.01, and *p* < 0.001 respectively. Superscripts a, b, and c indicate that the p value for the difference between cohorts is statistically significant at the 0.1%, 1%, and 5% levels, respectively. OPM poverty rate: 10.9 percent in 1979 cohort, 13.8 percent in 1997 cohort. All models control for age, missing poverty status when young, missing employment status, and missing educational attainment.

**Table B.3:** Estimated Effects of Benchmarks on Poverty Around Age 30; Alternative Specification of Partnering Before Children

	Model 1		Model 2		Model 3	
	1979	1997	1979	1979	1997	1979
Married before kids	−0.21***	−0.13***	−0.14***	−0.09***	−0.15***	−0.09***
	(0.03)	(0.01)	(0.03)	(0.01)	(0.03)	(0.01)
High school degree or more	−0.19***	−0.24***	−0.18***	−0.20***	−0.17***	−0.20***
	(0.02)	(0.02)	(0.02)	(0.02)	(0.02)	(0.02)
Consistent work	−0.13***	−0.12***	−0.12***	−0.12***	−0.12***	−0.12***
	(0.01)	(0.01)	(0.01)	(0.01)	(0.01)	(0.01)
Poverty when young			0.11***	0.13***	0.11***	0.13***
			(0.02)	(0.02)	(0.02)	(0.02)
Female			0.01	−0.02*	0.03**	−0.02
			(0.01)	(0.01)	(0.01)	(0.01)
Black			0.13***	0.15***	0.13***	0.15***
			(0.02)	(0.01)	(0.02)	(0.01)
Hispanic			0.05*	0.02	0.04*	0.01
			(0.02)	(0.01)	(0.02)	(0.01)
Living with parent(s) at age 30					0.08***	0.03**
					(0.02)	(0.01)
Number of children at age 30					−0.03*	−0.00
					(0.01)	(0.00)
Region of residence					X	X
Constant	0.77***	0.37*	0.51**	0.34*	0.40*	0.43*
	(0.15)	(0.17)	(0.16)	(0.17)	(0.16)	(0.18)
Observations	4822	6742	4822	6742	4822	6742

*Notes*: Authors' analyses using data from the 1979 and 1997 NLSY cohorts. Standard errors are presented in parentheses. The symbols *, **, and *** denote *p* < 0.05, p < 0.01, and *p* < 0.001 respectively. Superscripts a, b, and c indicate that the p value for the difference between cohorts is statistically significant at the 0.1%, 1%, and 5% levels, respectively. OPM poverty rate: 10.9 percent in 1979 cohort, 13.8 percent in 1997 cohort. All models control for age, missing poverty status when young, missing employment status, and missing education attainment.

## Footnotes

1 As a sensitivity check, we ran models using alternative measures of the three benchmarks of success—as described in the Measures sections above. The results from these models, provided in Appendix B, generally provide support for our main analysis. As with our main measure for education, we find the association between having at least some college, a bachelor's degree or more, or a post-bachelor's degree and reduced poverty have increased over time. We found no difference in the results from our analysis using the alternative measure for consistent employment compared to our main results. While our main models showed no change in the association between partnering before children and poverty across cohorts, results from our sensitivity check using an alternative measure for partnering before childbirth, show a four percentage point decrease in the association across cohorts (12 percentage points vs. 8 percentage points). As with our main models, differences across cohorts for all models were not statistically significant.
